# Geriatric Assessment in Patients Aged 70 Years and Older Considered for CAR T-Cell Therapy: A Retrospective Study

**DOI:** 10.3390/cancers18142289

**Published:** 2026-07-16

**Authors:** Anthony Tremblay, Rachel Boisvert, Manon Chevalier, Noémie Roux-Dubois, Nathalie Lambert-Perreault, Caroline Malenfant, Pierre-Hugues Carmichael, Jean-Philippe Émond, Christine Dionne

**Affiliations:** 1 VITAM—Centre de Recherche en Santé Durable, Québec, QC G1J 2G1, Canada; christine.dionne@fmed.ulaval.ca; 2 Centre de Recherche du CHU de Québec-Université Laval, Québec, QC G1S 4L8, Canada; noemie.roux-dubois.1@ulaval.ca (N.R.-D.); jean-philippe.emond.1@ulaval.ca (J.-P.É.); 3 Faculté de Médecine, Pavillon Ferdinand-Vandry, Université Laval, Québec, QC G1V 0A6, Canada; rachel.boisvert.2@ulaval.ca (R.B.); manon.chevalier@fmed.ulaval.ca (M.C.); 4 Département de Médecine, CHU de Québec, Québec, QC G1S 4L8, Canada; 5 Département de Médecine Spécialisée, CIUSSS de la Capitale-Nationale, Québec, QC G1S 4L8, Canada; 6 Direction du Programme Soutien à l’Autonomie des Personnes Âgées, CIUSSS de la Capitale-Nationale, Québec, QC G1S 4L8, Canada; nathalie.lambert-perreault.ciussscn@ssss.gouv.qc.ca; 7 Direction Médicale et des Services Professionnels, CIUSSS de la Capitale-Nationale, Québec, QC G1S 4L8, Canada; caroline.malenfant.ciussscn@ssss.gouv.qc.ca; 8 Centre d’Excellence sur le Vieillissement de Québec (CEVQ), Québec, QC G1S 4L8, Canada; pierre-hugues.carmichael.ciussscn@ssss.gouv.qc.ca

**Keywords:** geriatric assessment, CAR T-cell therapy, lymphoma, older adults, frailty, cytokine release syndrome, immune effector cell-associated neurotoxicity syndrome

## Abstract

**Background/Objectives**: Population aging has increased the incidence of non-Hodgkin lymphoma in older adults, who often receive suboptimal treatment. Geriatric assessment is a key tool to evaluate frailty and guide therapeutic decisions. CAR T-cell therapy is effective but associated with significant toxicities, including cytokine release syndrome (CRS) and immune effector cell-associated neurotoxicity syndrome (ICANS), which may be more severe in older patients. This study evaluated the role of geriatric assessments in patients aged 70 years and older referred for CAR T-cell therapy, focusing on patient profiles, treatment-related toxicities and clinical outcomes. **Methods**: This retrospective descriptive study included all patients aged 70 years and older for whom CAR T-cell therapy was considered at our institution between 15 December 2022 and 31 January 2026. Data were collected through medical record review. Descriptive and exploratory statistical analyses were performed. **Results**: A total of 43 patients aged 70 years or older with a diagnosis of refractory non-Hodgkin lymphoma underwent geriatric assessment prior to CAR T-cell therapy (median age: 73 years; 58% male). Most patients lived at home (98%), often with a cohabiting partner (65%). Polypharmacy (72%), mobility impairment (19%) and cognitive impairment (49%) were prevalent among our study population. Among the evaluated patients, CAR T-cell therapy was recommended in 35 cases, of whom 27 subsequently received treatment. In total, 85% developed CRS and 59% developed ICANS. ICANS occurred slightly more frequently in patients with pre-existing neurocognitive impairment (7 out of 11, 64%), compared with 9 out of 16 (56%) among those without cognitive impairment. The median length of hospitalization was 16 days. Mean overall survival post CAR T-cell therapy reached 324 days as of 31 January 2026 (median 277 days). Patients who were not recommended for CAR T-cell therapy exhibited a markedly higher burden of frailty compared with those who were and most of these vulnerabilities had not been identified before the geriatric evaluation. **Conclusions**: This study highlights the importance of oncogeriatric assessment in the context of CAR T-cell therapy to personalize treatment strategies according to patients’ frailty profiles. It may help optimize the care plan, potentially identifying patients most likely to benefit from therapy, while minimizing treatment-related toxicities. It also helps estimate the risk of treatment-related toxicities and facilitates the identification of previously unrecognized vulnerabilities.

## Simple Summary

Older adults with lymphoma are increasingly considered for CAR T-cell therapy, a highly effective but potentially toxic treatment. However, these patients are often underrepresented in clinical trials and treatment decisions are frequently based on age rather than a comprehensive evaluation of overall health status. This retrospective study examined the role of geriatric assessment in patients aged 70 years and older referred for CAR T-cell therapy. A substantial proportion of patients presented with previously unrecognized vulnerabilities, including cognitive impairment, polypharmacy and functional limitations. Patients who were not recommended for CAR T-cell therapy had a higher burden of frailty compared with those who were recommended. Among treated patients, treatment-related toxicities were common and neurotoxicity occurred numerically more often in patients with pre-existing cognitive impairment, although this finding was not statistically significant. The overall findings suggest that geriatric assessment helps identify patients most likely to benefit from CAR T-cell therapy and optimize their management.

## 1. Introduction

### 1.1. CAR T-Cell Therapy and Older Adults

Chimeric Antigen Receptor (CAR) T-cell therapy has emerged as a transformative and highly effective treatment modality for elderly patients with relapsed or refractory non-Hodgkin lymphoma, demonstrating that chronological age alone is not a valid parameter to guide curative-intent therapy [1,2,3]. Clinical data from multicenter studies and real-world registries indicate that patients aged 65 and older, including those in their 80s and 90s, can achieve objective response rates as high as 86% and complete response rates up to 62%, with efficacy outcomes comparable to younger cohorts and durable remissions even in ultra-elderly patients [2,4]. These findings indicate that the anti-tumor potency of CAR T-cells is largely preserved across age decades, provided that patients maintain sufficient physiologic reserve and functional capacity [2,4].

Despite these encouraging efficacy outcomes, older patients face distinct and clinically significant safety challenges. While the incidence of cytokine release syndrome (CRS) does not appear to differ significantly by age, increasing age is strongly associated with a higher incidence and severity of immune effector cell-associated neurotoxicity syndrome (ICANS), with patients aged ≥75 years exhibiting a hazard ratio of 2.532 compared to younger individuals [3]. Pre-existing neurologic and cognitive impairments may further increase the risk of severe neurotoxicity [2,4]. In parallel, cardiovascular toxicity represents a major concern in the geriatric population, with reported incidence rates of 28.7% compared to 13.5% in younger cohorts. These events, including arrhythmias and heart failure, are closely associated with CRS and necessitate intensive monitoring [5]. Non-relapse mortality appears to be predominantly driven by severe infectious complications related to profound post-treatment immunosuppression rather than age-related organ failure [2,5]. These risks highlight the need for rigorous pre-treatment screening and early intervention strategies in this higher-risk population.

In this context, there is growing consensus that functional status, rather than chronological age, is the primary determinant of outcomes in elderly patients undergoing CAR T-cell therapy [2,6]. Traditional metrics such as the Eastern Cooperative Oncology Group (ECOG) performance status remain clinically relevant, with scores ≥ 2 associated with inferior survival and increased toxicity [2]. However, more refined tools are required for optimal risk stratification in older adults. The Geriatric Prognostic Index (GPI), integrating activities of daily living, comorbidity burden assessed by the Charlson Comorbidity Index and nutritional parameters such as albumin levels, has demonstrated superior predictive performance compared to conventional indices like the International Prognostic Index in patients aged ≥70 years [6]. Geriatric assessments, including evaluations of cognitive function, gait speed, grip strength and instrumental activities of daily living, further allow clinicians to distinguish between fit and vulnerable patients, facilitating more precise therapeutic decision-making [4,6]. This paradigm is well illustrated in other complex, high-risk interventions frequently offered to older adults. In transcatheter aortic valve implantation (TAVI), frailty assessment has consistently been shown to predict mortality, procedural complications and functional decline beyond traditional risk scores [7,8,9]. As a result, frailty evaluation has progressively become an integral component of routine pre-procedural decision-making in this setting. A similar approach may be warranted for CAR T-cell therapy, where chronological age alone should not be used to exclude patients from potentially effective treatment, but should instead prompt a structured geriatric assessment to better estimate physiologic reserve, competing vulnerabilities and the likelihood of meaningful clinical benefit.

Beyond risk stratification, recent advances emphasize the importance of proactive patient optimization through multidisciplinary approaches. Geriatric assessment-guided multidisciplinary clinics (GA-MDC), involving close collaboration between hematologists, geriatricians, cardiologists, physical therapists and nutritionists, have emerged as a promising model of care [10]. These programs not only identify vulnerable patients but also implement targeted interventions to improve physical and nutritional status prior to leukapheresis. Notably, a clinical recommendation to proceed with CAR T-cell therapy following multidisciplinary evaluation has been shown to be an independent prognostic factor for overall survival, even after adjustment for biological markers such as lactate dehydrogenase and C-reactive protein [10]. Furthermore, this approach is associated with significantly reduced healthcare resource utilization, including shorter hospital stays and markedly lower rates of intensive care unit admissions.

Finally, optimizing outcomes in older adults requires a comprehensive strategy integrating appropriate product selection and early toxicity management [2,4]. High tumor burden, reflected by elevated lactate dehydrogenase levels and bulky disease, remains a key adverse prognostic factor regardless of age [2]. The selection of CAR T-cell products with more favorable safety profiles, such as lisocabtagène maraleucel, may be particularly relevant in frail or very elderly patients due to a lower incidence of severe CRS and ICANS [2,4]. Ultimately, the combination of individualized risk assessment, proactive geriatric optimization and multidisciplinary care is essential to maximizing both efficacy and safety in this rapidly expanding patient population.

### 1.2. Objective

The present study aims to explore the role of geriatric assessment in the context of CAR T-cell therapy among older adults.

The first objective is to analyze the geriatric assessment process conducted in patients referred to the Quebec Oncogeriatric Clinic prior to CAR T-cell therapy. This objective seeks to describe the multidimensional domains evaluated, as well as the diagnoses established by the oncogeriatrician and the recommendations formulated. It also aims to document how these recommendations influenced overall patient management and modified the initial therapeutic plan.

The second objective is to establish a detailed clinical profile of older adults evaluated prior to CAR T-cell therapy. This profile will allow for precise characterization of this patient population.

The third objective is to describe the frequency and severity of the principal toxicities associated with CAR T-cell therapy, namely, CRS, ICANS and febrile neutropenia. This includes examining potential associations between toxicities and frailty-related vulnerabilities to determine whether specific patient profiles are at increased risk of complications.

The last objective is to describe patients’ clinical trajectories following geriatric assessment. Outcomes of interest include whether the patient subsequently received the recommended therapy, length of hospitalization, overall survival and mortality. This objective aims to provide a comprehensive overview of the care pathway and to evaluate the impact of geriatric assessment on clinical outcomes.

## 2. Materials and Methods

This retrospective descriptive study included all patients aged 70 years and older with refractory non-Hodgkin lymphoma at our institution for whom CAR T-cell therapy was considered between 15 December 2022 and 31 January 2026. At our center, all patients meeting these criteria were systematically referred for geriatric assessment. Patients underwent a geriatric assessment at the Oncogeriatrics Clinic of Hôpital du Saint-Sacrement, part of the Centre Intégré Universitaire de Santé et de Services Sociaux de la Capitale-Nationale (CIUSSSCN) in Québec City, Canada. This clinic is a tertiary referral center in oncogeriatrics covering the eastern part of the Province of Québec. No exclusion criteria were applied. Data were collected through retrospective review of patients’ medical records. Descriptive statistical analyses were performed and results are reported using means, standard deviations and proportions where appropriate. Missing data were handled using a complete-case approach, with analyses restricted to available observations for each variable. No imputation methods were used. Missing data are reported in the tables by indicating the number of available observations (*n*/*N*) for each variable. Baseline variables were compared according to the oncogeriatrician’s recommendation and actual receipt of CAR T-cell therapy using Fisher’s exact tests for categorical variables and Wilcoxon rank-sum tests for continuous variables. These analyses were exploratory and descriptive. They were intended to compare patient characteristics between groups and were not designed to identify independent factors associated with the oncogeriatrician’s recommendation or receipt of CAR T-cell therapy. Given the limited sample size, particularly the small number of patients for whom CAR T-cell therapy was not recommended, multivariable analyses were not performed. All statistical analyses were performed using R software (version 4.6.1; R Core Team, Vienna, Austria).

Comorbidity burden was quantified using the Charlson Comorbidity Index. Functional status was assessed through independence in activities of daily living (ADL) and instrumental activities of daily living (IADL). Cognitive function was evaluated using the Montreal Cognitive Assessment (MoCA). For the nutritional status, the presence of decreased appetite, weight loss and malnutrition were noted. Mobility and physical performance measures included gait speed and grip strength.

The presence of selected geriatric syndromes was documented. Polypharmacy was defined as the use of five or more different medications [11], excluding laxatives and vitamins. The presence of cognitive impairment, malnutrition and mobility impairment were determined based on the oncogeriatrician’s evaluation.

The oncogeriatrician’s recommendations were collected, including recommendations for CAR T-cell treatment and referrals to allied health professionals.

Follow-up data were collected from the date of oncogeriatric assessment until death or the data cutoff date of 31 January 2026. For all patients, follow-up variables included receipt of CAR T-cell therapy, mortality status and overall survival. Among patients who received CAR T-cell therapy, additional data were collected on the type of CAR T-cell product administered, post-CAR T-cell length of hospitalization and the occurrence of CRS, ICANS and febrile neutropenia. Overall survival was defined as the time from oncogeriatric assessment to death from any cause and was estimated using the Kaplan–Meier method, with censoring at the data cutoff date.

The following describes the assessment process at our clinic. When patients are referred to the Québec Oncogeriatric Clinic, they undergo a geriatric evaluation beginning with a consultation conducted by a clinical nurse. This initial assessment includes a detailed evaluation of the patient’s cognitive, functional and psychosocial profile. The nurse interviews the patient and a close relative regarding independence in activities of daily living and instrumental activities of daily living, as well as mobility status. Information is also gathered regarding lifestyle habits, medication use, living environment, transportation availability and the presence of a caregiver. Nutritional habits, sleep quality and affective symptoms are explored and screening is performed using the Geriatric Depression Scale (GDS) when appropriate [12]. Objective cognitive evaluation is conducted using the Montreal Cognitive Assessment (MoCA). Vital signs, gait speed, grip strength in both hands and anthropometric measurements (height and weight) are recorded as well.

The second phase of the evaluation involves consultation with the oncogeriatrician. The physician reviews the medical record, documenting known comorbidities and current medications. Based on the information collected by the nurse, the oncogeriatrician meets the patient and accompanying relative, performs a physical examination and discusses the goal of the consultation. The patient’s understanding of their medical condition and proposed therapeutic options is assessed in order to evaluate decision-making capacity. The oncogeriatrician also explores the patient’s goals of care, life priorities and values, as well as the potential risks of proposed treatments and their impact on cognitive and physical function. Level-of-care preferences may also be discussed.

Then, the oncogeriatrician formulates recommendations and discusses with the referring hematologist-oncologist. In our clinic, these recommendations are followed in 100% of cases of pre-CAR T-cell evaluation.

## 3. Results

### 3.1. Patient Flow and Characteristics of the Overall Study Population

**Figure 1:**
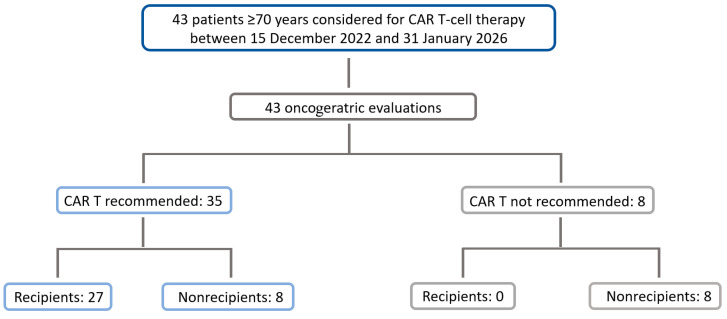
Flow diagram of patients aged ≥70 years considered for CAR T-cell therapy. Figure includes all older adults who were considered for CAR T-cell therapy between 15 December 2022 and 31 January 2026 at the CHU de Québec. All patients underwent an oncogeriatric assessment.

From 15 December 2022 to 31 January 2026, 43 patients aged 70 years and older who were being considered for CAR T-cell therapy at the CHU de Québec underwent an oncogeriatric assessment. The oncogeriatrician recommended proceeding with CAR T-cell therapy in 35 patients, of whom 27 had received the treatment by the data cutoff date of 31 January 2026. Among the remaining patients, one declined treatment, five died before therapy initiation and two had not yet received treatment at the time of data cutoff. The oncogeriatrician recommended against CAR T-cell therapy in 8 patients and none of them received the treatment. Figure 1 presents a flow chart of the patients evaluated.

### 3.2. Demographics, Disease Characteristics and Recommendation Decision in Older Adult Candidates for CAR T-Cell Therapy Evaluated by the Oncogeriatrician

Table 1 summarizes the demographic characteristics, disease characteristics and oncogeriatric recommendation for the study population of older adults considered for CAR T-cell therapy.

In the overall cohort, the median age was 73 years and 58% of patients were men. Most patients lived at home (98%), whereas only one patient lived in a seniors’ residence. Overall, 65% of patients were living with someone. Compared with patients for whom CAR T-cell therapy was recommended, those for whom the oncogeriatrician recommended against CAR T-cell therapy were slightly older (75 vs. 73 years), were more frequently women (62% vs. 37%) and were less likely to be living with someone (25% vs. 74%).

Among patients who ultimately received CAR T-cell therapy, compared with those who did not, the median age was lower (72 vs. 75 years), a higher proportion lived at home (100% vs. 94%) and with someone (74% vs. 50%). The sex distribution was similar between these two groups.

When comparing patients who were recommended for CAR T-cell therapy with those who were not, cohabitation status differed significantly between groups (*p* < 0.05). In addition, among patients who ultimately received CAR T-cell therapy compared with those who did not, age was significantly different between groups (*p* < 0.05). However, given the retrospective design and limited sample size, these statistically significant differences should be interpreted with caution. Their clinical significance remains uncertain and these findings should be considered exploratory rather than definitive.

### 3.3. Geriatric Assessment

Table 2 presents the baseline geriatric assessment findings for the 43 patients evaluated. All patients were followed by a hematologist-oncologist. In the overall cohort, the median weight was 79 kg and the height was 170 cm. Median grip strength was within the expected range for age, with values of 25 kg for the left hand and 26 kg for the right hand. Median gait speed and MoCA score were also within the expected range, at 1.17 m/s and 25, respectively. The median Charlson Comorbidity Index was 6, reflecting a substantial comorbidity burden in the study population. More specifically, 23% of patients had a score of 5, 33% a score of 6, 23% a score of 7, 14% a score of 8 and 7% a score of 9. The number of medications was also high, with a median of 6 medications per patient.

From a nutritional perspective, 42% of patients reported weight loss in the previous 3 months and 23% reported decreased appetite. Most patients were fully independent in their activities of daily living (ADL) and instrumental activities of daily living (IADL) (67%). However, 30% had at least one IADL dependency and 2% had IADL and ADL dependency. A total of 23% of patients reported at least one fall in the previous year. In addition, 16% of patients were referred to another healthcare professional to address frailty-related vulnerabilities identified during the geriatric assessment.

When comparing patients for whom the oncogeriatrician recommended CAR T-cell therapy with those for whom treatment was not recommended, the latter group showed a less favorable geriatric profile. Patients in the group not recommended for CAR T-cell therapy had a lower median weight (69 kg vs. 81 kg), lower median grip strength (14 kg vs. 26 kg for the left hand and 15 kg vs. 28 kg for the right hand), slower median gait speed (1.1 m/s vs. 1.2 m/s) and a lower median MoCA score (22 vs. 25). They also had a higher median Charlson Comorbidity Index (7 vs. 6), a higher prevalence of weight loss (65% vs. 35%), decreased appetite (50% vs. 17%), ADL dependency (13% vs. 0%), IADL dependency (50% vs. 26%) and at least one fall in the previous year (38% vs. 20%). Referrals to other healthcare professionals were also more frequent in this group (38% vs. 11%). The median number of medications was similar between groups, with 6 medications. A similar pattern was observed when comparing patients who ultimately received CAR T-cell therapy with those who did not.

Several geriatric assessment domains differed significantly between patients who were recommended for CAR T-cell therapy and those who were not. Statistically significant differences were observed for right-hand grip strength, gait speed, MoCA score, malnutrition, autonomy, cognitive disorder, mobility impairment, mood disorder and social vulnerability. When comparing patients who ultimately received CAR T-cell therapy with those who did not, statistically significant differences were limited to functional independence, cognitive disorder and mobility impairment. These findings suggest that selected geriatric parameters differed across treatment decision groups, although they should be interpreted cautiously given the small sample size and the exploratory nature of these comparisons.

Frailty-related vulnerabilities were frequent in our cohort, particularly among patients for whom the oncogeriatrician recommended against proceeding with CAR T-cell therapy. Most of these diagnoses were newly identified during the oncogeriatric assessment and had not been previously documented.

In the overall cohort, social vulnerabilities were identified in 21% of patients, polypharmacy in 72%, cognitive impairment in 49%, mobility impairment in 19%, malnutrition in 30% and mood disorders in 5%. Overall, 49% of patients had a neurocognitive disorder, including 35% with a mild neurocognitive disorder and 14% with a major neurocognitive disorder. Specific recommendations were formulated in most cases, including fall prevention strategies, medication optimization, nutritional follow-up and cognitive stimulation.

**Figure 2:**
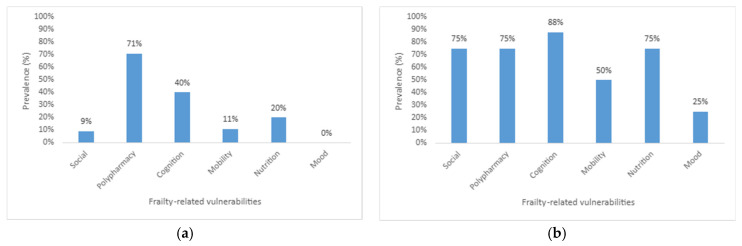
Prevalence of frailty-related vulnerabilities according to the oncogeriatrician’s recommendation. (**a**) Patients for whom CAR T-cell therapy was recommended (*n* = 35). (**b**) Patients for whom CAR T-cell therapy was not recommended (*n* = 8).

Frailty-related vulnerabilities were more frequent among patients for whom CAR T-cell therapy was not recommended. Figure 2 compares the prevalence of frailty-related vulnerabilities according to whether CAR T-cell therapy was recommended.

Among the 43 patients evaluated, the oncogeriatrician recommended against proceeding with CAR T-cell therapy in 8 patients. Among these, cognitive vulnerability was the most frequently documented reason, being cited in 7 cases. Lack of capacity to provide informed consent was documented in 3 patients. Nutritional, mobility and social vulnerabilities were also frequently reported. Table 3 presents the reasons documented for each of these 8 patients.

### 3.4. Clinical Outcomes and Toxicities in Patients Treated with CAR T-Cell Therapy

Table 4 presents the characteristics of the 27 (63%) patients who received CAR T-cell therapy. Axicabtagène ciloleucel (Yescarta; Kite Pharma Inc., Santa Monica, CA, USA) was the most frequently administered CAR T-cell therapy (67%), followed by brexucabtagène autoleucel (Tecartus; Kite Pharma Inc., Santa Monica, CA, USA) and lisocabtagène maraleucel (Breyanzi; Bristol Myers Squibb Inc., Princeton, NJ, USA), which were administered in equal proportions (15% each). Allogeneic CAR T-cell therapy was administered in 1 patient. Among the 27 patients, 23 (85%) developed CRS, including 14 grade 1 cases (52%) and 9 grade 2 cases (33%). No grade 3 or 4 CRS events were observed. ICANS occurred in 16 patients (59%), including 6 patients (22%) with grade ≥ 2 events. More specifically, 10 patients had grade 1 (37%), 3 had grade 2 (11%), 2 had grade 3 (7%), and 1 had grade 4 (4%). Among the 11 patients with cognitive disorder who received CAR T-cell therapy, 7 (64%) developed ICANS, compared with 9 (56%) among those without cognitive impairment. Febrile neutropenia occurred in 16 patients (59%). The median length of hospitalization following treatment was 16 days (SD 11.26).

At the data cutoff date (31 January 2026), the mean overall survival after CAR T-cell therapy was 324 days (SD 275.85) and the median was 277 days. Nine patients (33%) had died following CAR T-cell therapy, with a mean overall survival of 299 days prior to death (SD 274.69) in this subgroup.

**Figure 3:**
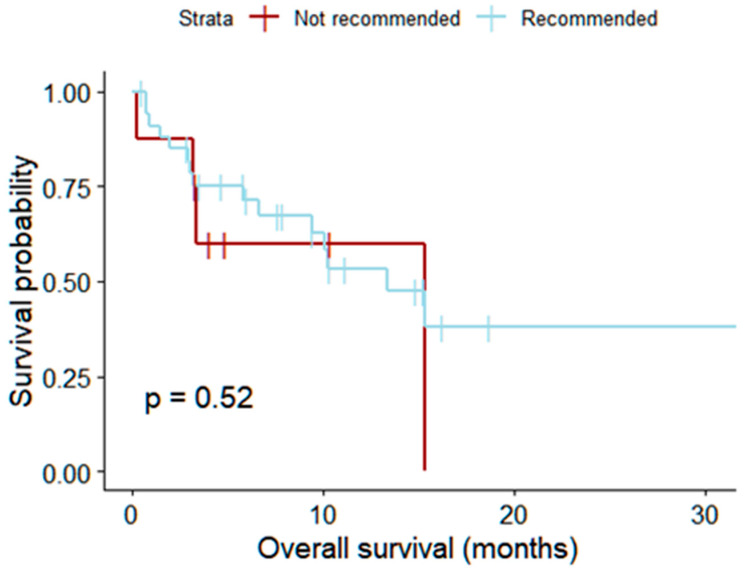
Overall survival from the date of oncogeriatric assessment according to oncogeriatrician recommendation for CAR T-cell therapy.

Figure 3 presents the Kaplan–Meier overall survival curves according to the oncogeriatrician’s recommendation regarding CAR T-cell therapy, calculated from the date of oncogeriatric assessment. Overall, survival trajectories appeared similar between patients for whom CAR T-cell therapy was recommended and those for whom it was not, with no statistically significant difference between groups (*p* = 0.52). In the recommended group, the survival curve appeared to plateau during longer follow-up, suggesting that some patients experienced prolonged survival.

## 4. Discussion

In this observational study, the profile of patients undergoing an oncogeriatric evaluation prior to CAR T-cell therapy was described. Differences between patients who were estimated to be fit for treatment after geriatric assessment and those for whom treatment was not recommended were documented, demonstrating a higher prevalence of geriatric syndromes in the second group.

Recent evidence suggests that CAR T-cell therapy can be safely and effectively administered in selected older adults, with outcomes comparable to those observed in younger populations, despite a higher burden of comorbidities and frailty [13,14]. However, chronological age alone remains a poor predictor of treatment tolerance and increasing attention has been directed toward the role of frailty and geriatric vulnerabilities in shaping outcomes [15,16]. Emerging studies have demonstrated that geriatric assessments may help identify patients at higher risk of treatment-related toxicities and adverse outcomes, while also uncovering previously unrecognized vulnerabilities [17,18]. In this context, our study contributes to the growing body of literature by further characterizing the role of pre-treatment oncogeriatric assessment in guiding patient selection and optimizing care in older adults considered for CAR T-cell therapy. This is particularly important as CAR T-cell therapy is increasingly being offered to older adults [19].

This study sought to better understand the role of geriatric assessment in the management of this population, which remains underrepresented in clinical trials. Our findings support the relevance of this approach, demonstrating that pre-treatment geriatric assessment provides a comprehensive understanding of overall health status beyond traditional oncologic criteria (such as the ECOG). The recommendations were consistently followed by the oncologist, contributing meaningfully to guiding the care trajectory.

For the first objective, analysis of the geriatric assessment process highlighted the complexity of patient profiles and the multidimensional nature of the evaluation, encompassing functional, cognitive, nutritional and social domains. Recommendations focused on fall prevention, medication optimization, maintenance of independence at home, prevention of cognitive and functional decline, and nutritional support translated into concrete adjustments in patient management. These observations reinforce the notion that geriatric assessment extends beyond patient selection. It functions as a therapeutic optimization tool and a catalyst for geriatric co-management.

For the second objective, the sociodemographic and medical profile of the patients illustrates the marked heterogeneity among older adults considered for CAR T-cell therapy. Despite a median age of 73 years and the presence of multiple comorbidities, patients exhibited substantial variability in functional, cognitive and clinical status, with some remaining highly independent and others presenting significant frailty. This heterogeneity shows that older adults represent a highly diverse population, in whom chronological age alone is insufficient to capture health status and underscores the need for individualized assessment rather than age-based decisions. However, chronological age may serve as a practical screening tool to identify patients in whom a geriatric assessment is indicated. In our study, we selected a threshold of 70 years to perform a geriatric assessment. Although this cutoff is arbitrary, it reflects the well-recognized physiological changes that occur with advancing age, particularly beyond 70 years [20], which may affect tolerance to complex cancer therapies. Importantly, our findings demonstrated a high prevalence of previously unrecognized geriatric vulnerabilities in this population, further supporting the relevance of systematic assessment. These observations are consistent with and reinforce current recommendations from the International Society of Geriatric Oncology (SIOG) and the European Society for Medical Oncology (ESMO), which advocate for the use of geriatric assessment in all patients aged 70 years and older (65 years and older when possible) undergoing cancer treatment [21]. As clinical trials frequently exclude frail individuals, a gap is being created between research and real-world practice [22,23]. Future studies should address this issue and examine if the cutoff age of 70 years is optimal to determine the need for a geriatric assessment in the pre-CAR T-cell setting.

For the third objective, the frequency and severity of CRS in our population were comparable to those reported in younger cohorts (85% vs. 57–93%) [24]. This observation supports the hypothesis that frailty identified and addressed prior to treatment may mitigate the risk of severe complications such as CRS. It also suggests that oncogeriatric assessment helps identify patients who are most likely to tolerate CAR T-cell therapy. However, the incidence of ICANS in our cohort appears higher than that reported in studies involving younger populations. In our study, ICANS occurred in 59% of patients, predominantly grade 1–2 toxicities, whereas a recent meta-analysis reported an incidence of 26.9% [25]. This may be attributable to the high prevalence of neurocognitive disorders in our treated population (41%). Only three patients (11%) developed grade ≥ 3 ICANS, which is comparable to the 10.5% reported in the meta-analysis [25]. These findings should be interpreted with caution given the small number of events.

Within the limits of a descriptive analysis and the limited sample size, ICANS appeared numerically more frequent among patients with cognitive disorder, even when mild. However, this association was not statistically significant in our cohort (*p* = 1.00), likely reflecting limited statistical power, as only 27 patients received CAR T-cell therapy, among whom 11 had a pre-existing neurocognitive disorder. This finding should therefore be interpreted as exploratory and hypothesis-generating. Nevertheless, this hypothesis is biologically plausible and supported by evidence suggesting that pre-existing neurocognitive impairment and neurologic comorbidities may influence neurotoxicity outcomes following CAR T-cell therapy through reduced cognitive reserve and increased vulnerability to inflammatory brain injury. The findings remain inconsistent and the association with ICANS has not been definitively established [26,27,28]. Pre-existing neuroinflammatory states and polypharmacy may further contribute to reduced tolerance of neurotoxic effects associated with cellular therapies [29]. These considerations support the importance of systematic cognitive evaluation prior to treatment initiation to better anticipate and manage CAR T-cell-related neurological complications. One limitation in interpreting these findings is the occurrence of delirium, which can be clinically difficult to distinguish from ICANS. Regarding febrile neutropenia, its incidence was slightly lower in our cohort (59%) compared with rates reported in the literature (77%) [30], likely related to the early administration of G-CSF in cases of neutropenia in our center [31].

For the fourth objective, the mean length of hospitalization was 20 days and the median was 16 days, which is similar to the median duration of 15 days reported in the literature [32]. Mean overall survival after CAR T-cell therapy administration among the 27 treated patients was 324 days at the cutoff date (median of 277 days). At this time point, 10 patients (37%) had survived beyond 365 days following CAR T-cell therapy, while several others had received treatment closer to the cutoff date, limiting the ability to fully assess 12-month overall survival despite favorable early clinical evolution. In the literature, median overall survival post CAR T-cell therapy among patients aged 65 years and older has been reported at 17.1 months [14], whereas median survival in our cohort was 9.1 months (277 days). However, this comparison should be interpreted with caution. In our study, survival was calculated between the first oncogeriatric consultation and a fixed data cutoff date of 31 January 2026 and patients did not all initiate treatment at the same time. As a result, follow-up duration varied across patients, with some having a shorter observation period. Moreover, longitudinal data specifically describing survival outcomes in older adults receiving CAR T-cell therapy remain limited, making direct comparisons with published cohorts challenging.

Five patients deemed eligible for CAR T-cell therapy died before treatment initiation, often following the occurrence of medical complications, rapid functional or clinical decline. This underscores both the aggressiveness of refractory disease and the potentially prolonged time to treatment initiation [33,34]. It also reinforces this study’s premise that early geriatric assessment may help identify patients at risk of not proceeding to treatment and facilitate proactive reorientation of care goals. Importantly, geriatric evaluations in our setting were completed within two weeks of referral and prior to the scheduled leukapheresis, therefore not delaying treatment initiation.

When comparing patients for whom CAR T-cell therapy was recommended with those for whom it was not, we observed a substantially higher burden of frailty among patients for whom the oncogeriatrician did not recommend therapy. Importantly, a significant proportion of these vulnerabilities had not been identified during the initial assessment by the referring team. The systematic integration of geriatric assessment prior to CAR T-cell therapy enables structured identification and grading of frailty-related factors, and facilitates the implementation of corrective interventions when appropriate. This approach not only supports assessment of treatment appropriateness but also enhances anticipation of tolerance and potential complications. Our findings therefore suggest that geriatric assessment is an important tool in identifying older patients most likely to benefit from CAR T-cell therapy.

The statistically significant differences observed across several geriatric domains may provide insight into the factors considered by oncogeriatricians when assessing eligibility for CAR T-cell therapy. Functional status, cognition, mobility, nutritional status, mood and social vulnerability differed between patients who were recommended for CAR T-cell therapy and those who were not, suggesting that the recommendation process was based on a multidimensional appraisal rather than chronological age alone. However, these findings must be interpreted with caution because of the limited cohort size, the retrospective design and the uncertain clinical significance of some statistically significant *p* values. In addition, for some variables, statistical significance was observed despite sparse data or the absence of events in one comparison group, notably for functional independence and cognitive disorder. Therefore, these results should be considered hypothesis-generating and descriptive, rather than definitive evidence of the relative weight of each geriatric domain in clinical decision-making.

The oncogeriatric team recommended against CAR T-cell therapy in eight patients, mainly because of cognitive impairment, inability to provide informed consent and other frailty-related vulnerabilities identified during the geriatric assessment. This finding highlights the value of a systematic oncogeriatric evaluation in patients aged 70 years and older who are being considered for CAR T-cell therapy. Such an assessment helps identify patients at high risk of treatment-related complications or those unlikely to derive meaningful clinical benefit, thereby avoiding potentially harmful therapy. This is particularly relevant in healthcare systems with limited resources, where highly specialized and costly treatments such as CAR T-cell therapy may place substantial pressure on the system without necessarily improving outcomes for some patients. Early identification of these vulnerable patients also allows clinicians to promptly redirect care toward alternative oncologic strategies, supportive care, or end-of-life care when appropriate. This issue is especially important in patients with pre-existing neurocognitive disorders, who may be at increased risk of clinically significant decline and ICANS. In these patients, even when lymphoma control is achieved, the competing trajectory of neurodegenerative disease may limit the overall benefit of treatment, particularly if CAR T-cell therapy contributes to accelerated cognitive or functional deterioration.

A recent study [10] evaluated the role of a geriatric assessment-guided multidisciplinary clinic in 61 patients being considered for CAR T-cell therapy. Direct comparison with our study is limited by several important differences. First, their cohort included some patients younger than 70 years, whereas our study focused exclusively on patients aged 70 years and older. Second, not all eligible patients were evaluated in their geriatric assessment-guided clinic, while systematic referral was implemented in our cohort. Third, their population included patients with hematologic malignancies other than non-Hodgkin lymphoma, including multiple myeloma, and therefore included both CD19- and BCMA-directed CAR T-cell products. Finally, their multidisciplinary clinic could recommend deferral before treatment and the recommendation was not mandatory, as some patients received CAR T-cell therapy despite a recommendation against proceeding.

Despite these differences, several findings were consistent between the two studies. The incidence of CRS was almost identical, occurring in 84.9% of patients in their cohort and in 85% of patients in ours. Any-grade ICANS was slightly more frequent in our cohort than in theirs (59% vs. 47.2%); however, this difference appeared to be driven mainly by grade 1 events. In contrast, grade ≥ 2 ICANS was less frequent in our cohort (22% vs. 32.1%). This may reflect differences in patient selection and the fact that patients in whom CAR T-cell therapy was not recommended by the oncogeriatrician did not receive treatment in our study. In the compared study, the main reasons for deferral or non-recommendation were predominantly related to physical function, nutrition, comorbidities, caregiver support, infection, or mood disorders, whereas cognitive impairment and inability to provide informed consent were central factors in the oncogeriatric recommendation in our cohort. This may have helped avoid exposing patients with significant neurocognitive vulnerability to CAR T-cell therapy, potentially limiting the occurrence of clinically significant ICANS. The median length of hospitalization was also similar between the two studies, at 17 days in their study and 16 days in our cohort.

Although overall survival did not differ significantly according to the oncogeriatrician’s recommendation in our cohort, this finding should be interpreted with caution. In contrast, Yates et al. [10] reported that a geriatric assessment-guided recommendation was associated with overall survival after CAR T-cell therapy. The absence of a statistically significant difference in our study may be explained by the smaller sample size, the limited number of patients in the non-recommended group and the absence of patients who received CAR T-cell therapy despite a recommendation against proceeding. In addition, survival was calculated from the date of oncogeriatric assessment and patients who did not proceed to CAR T-cell therapy followed heterogeneous clinical trajectories, including rapid clinical deterioration, death before treatment initiation, alternative oncologic strategies or supportive care. These factors limit direct comparison between groups and support interpreting this analysis as exploratory.

This study nevertheless has several limitations. The limited sample size and single-center design, coupled with retrospective data collection, limit statistical power while reducing generalizability. Together, these limitations precluded robust multivariable analyses to identify independent factors associated with the oncogeriatrician’s recommendation or receipt of CAR T-cell therapy. Observed differences between groups should therefore be interpreted as descriptive and exploratory rather than as evidence of independent associations. Although some observed differences did not reach statistical significance, likely because of limited power, they may remain clinically relevant and help inform practice in this specific population. In addition, we did not include a comparison cohort, which limits our ability to demonstrate that geriatric assessment directly improves survival or reduces treatment-related toxicities. Some important oncologic variables, including performance status, prior lines of therapy and disease burden were not systematically collected, limiting our ability to adjust outcomes for key prognostic factors. Survival analyses should also be interpreted with caution. Follow-up was limited and immature, with a fixed data cutoff date and some patients had short follow-up because CAR T-cell therapy was administered close to the cutoff date. However, our findings provide a foundation for future prospective studies aimed at further systematically exploring the relationship between frailty markers, post-CAR T-cell toxicity, healthcare utilization and overall survival in older adults considered for CAR T-cell therapy.

## 5. Conclusions

This study highlights the potential role of geriatric assessment in older adults considered for CAR T-cell therapy. Oncogeriatric consultation enables the identification of comorbidities and frailty-related vulnerabilities, allowing for a thorough evaluation of treatment appropriateness and, to some extent, a more accurate estimation of adverse events such as ICANS.

This approach facilitates individualized treatment planning and optimization of the care trajectory while promoting the preservation of functional autonomy and quality of life. Our findings support the systematic integration of oncogeriatric assessment into cellular therapy pathways for patients aged 70 years and older to ensure safer, more personalized and older adult-centered care. Larger prospective studies are needed to confirm these observations, clarify the optimal age threshold for assessment, refine patient selection for referral to oncogeriatric clinics and further characterize the relationship between pre-existing neurocognitive disorders and the development of ICANS.

## Figures and Tables

**Table 1:** Demographics, disease characteristics and recommendation decision in older adult candidates for CAR T evaluated by the oncogeriatrician.

		Oncogeriatrician Recommendation			Receipt of CAR T-Cell Therapy			
Characteristic	All Patients (*N* = 43)	Recommended (*n* = 35)	Not Recommended (*n* = 8)	*p* Value	CAR T Recipient (*n* = 27)	CAR T Nonrecipients (*n* = 16)	*p* Value	Data Available (*n*/*N*)
Age (years), median (IQR)	73 (71.5, 76)	73 (71, 76)	75 (73, 76.3)	0.265	72 (71, 75.5)	75 (73, 77.3)	0.015	43/43
Male sex, *n* (%)	25 (58%)	22 (63%)	3 (38%)	0.247	16 (59%)	9 (56%)	1.000	43/43
Living situation, *n* (%)				1.000			0.372	43/43
Home	42 (98%)	34 (97%)	8 (100%)		27 (100%)	15 (94%)		
Senior residence	1 (3%)	1 (3%)	0 (0%)		0 (0%)	1 (6%)		
Cohabitation, *n* (%)	28 (65%)	26 (74%)	2 (25%)	0.014	20 (74%)	8 (50%)	0.185	43/43
Diagnosis, *n* (%)				0.416			0.586	43/43
DLBCL	34 (79%)	26 (74%)	8 (100%)		20 (74%)	14 (88%)		
Mantle cell	7 (16%)	7 (20%)	0 (0%)		5 (19%)	2 (12%)		
Follicular	2 (5%)	2 (6%)	0 (0%)		2 (7%)	0 (0%)		

**Table 2:** Results of baseline geriatric assessments in older adults under consideration for CAR T-cell therapy.

		Oncogeriatrician Recommendation			Receipt of CAR T-Cell Therapy			
Characteristic	All Patients (*N* = 43)	Recommended (*n* = 35)	Not Recommended (*n* = 8)	*p* Value	CAR T Recipients (*n* = 27)	CAR T Nonrecipients (*n* = 16)	*p* Value	Data Available (*n*/*N*)
Weight (kg), median (IQR)	79 (67.3, 91.2)	81 (69.2, 92.7)	69 (47.9, 88)	0.151	76 (69.2, 94)	81 (59, 88.3)	0.466	43/43
Height (cm), median (IQR)	170 (166.3, 175.4)	170 (167, 175)	165 (154, 179.8)	0.841	170 (167, 175)	174 (166, 178)	0.632	38/43
Grip strength (kg), median (IQR)								35/43
Left	25 (16.2, 35.2)	26 (18.8, 36.7)	14 (13.8, 24.2)	0.083	25 (18.8, 34.6)	18 (14.3, 35.33)	0.5	
Right	26 (18.6, 36.3)	28 (22.3, 37.7)	15 (11.2, 22)	0.013	27 (22.3, 36.8)	19 (11.18, 32.8)	0.118	
Gait speed (m/s), median (IQR)	1.2 (1.1, 1.3)	1.2 (1.1, 1.3)	1.1 (1, 1.2)	0.048	1.2 (1.1, 1.3)	1.2 (1, 1.2)	0.324	34/43
MoCA, median (IQR)	25 (23, 26.5)	25 (23.5, 27)	22 (17.3, 24)	0.017	26 (23.5, 26.5)	24 (22, 25.5)	0.114	43/43
Charlson Comorbidity Index, median (IQR)	6 (6, 7)	6 (5.5, 7)	7 (6.8, 7.3)	0.152	6 (5, 7)	7 (6, 8)	0.113	43/43
Number of drugs, median (IQR)	6 (4, 8)	6 (4, 8)	6 (4.5, 9.3)	0.814	5 (4, 7.5)	7 (5, 8.3)	0.25	43/43
Polypharmacy (≥5 medications), *n* (%)	31 (72%)	25 (71%)	6 (75%)	1	17 (63%)	14 (88%)	0.158	43/43
Weight loss in the past 3 months, *n* (%)	18 (42%)	13 (37%)	5 (63%)	0.247	9 (33%)	9 (56%)	0.204	43/43
Malnutrition, *n* (%)	13 (30%)	7 (20%)	6 (75%)	0.006	6 (22%)	7 (44%)	0.178	43/43
Decreased appetite, *n* (%)	10 (23%)	6 (17%)	4 (50%)	0.07	4 (15%)	6 (38%)	0.137	43/43
Autonomy, *n* (%)				0.038			0.022	43/43
Completely independent	29 (67%)	26 (74%)	3 (38%)		22 (82%)	7 (44%)		
Dependent IADL and ADL, *n* (%)	1 (2%)	0 (0%)	1 (13%)		0 (0%)	1 (6%)		
Dependent IADL, *n* (%)	13 (30%)	9 (26%)	4 (50%)		5 (19%)	8 (50%)		
≥1 fall in the last year, *n* (%)	10 (23%)	7 (20%)	3 (38%)	0.362	6 (22%)	4 (25%)	1	43/43
Cognitive disorder, *n* (%)				<0.001			0.003	43/43
Minor	15 (35%)	14 (40%)	1 (13%)		11 (41%)	4 (25%)		
Major	6 (14%)	0 (0%)	6 (75%)		0 (0%)	6 (38%)		
Mobility impairment, *n* (%)	8 (19%)	4 (11%)	4 (50%)	0.028	2 (7%)	6 (38%)	0.037	43/43
Mood disorder, *n* (%)	2 (5%)	0 (0%)	2 (25%)	0.031	0 (0%)	2 (13%)	0.133	
Social vulnerability, *n* (%)	9 (21%)	3 (9%)	6 (75%)	<0.001	3 (11%)	6 (38%)	0.058	
Referral to a health professional, *n* (%)	7 (16%)	4 (11%)	3 (38%)	0.106	3 (11%)	4 (25%)	0.394	43/43

**Table 3:** Reasons underlying the recommendation against CAR T-cell therapy (*N* = 8).

Patient	Reasons for Non-Recommendation
1	Patient’s goals of care Comorbidities Frailty: Nutrition, mobility
2	Lack of capacity to consent to care Frailty: Cognition, nutrition
3	Lack of capacity to consent to care Comorbidities Frailty: Cognition, mobility, social
4	Lack of capacity to consent to care Frailty: Cognition
5	Frailty: Cognition
6	Frailty: Cognition, mobility, social, mood, polypharmacy
7	Frailty: Cognition, nutrition, social, mood, polypharmacy
8	Frailty: Cognition, nutrition, mobility, social

**Table 4:** CAR T-cell therapy administered and outcomes (*N* = 27).

Characteristic	Value	Standard Deviation	Data Available (*n*/*N*)
Type of CAR T received, *n* (%)			27/27
Axicabtagène ciloleucel	18 (67%)		
Brexucabtagène autoleucel	4 (15%)		
Lisocabtagène maraleucel	4 (15%)		
Allogeneic	1 (4%)		
Cytokine release syndrome (CRS), *n* (%)	23 (85%)		27/27
Grade 1	14 (52%)		
Grade 2	9 (33%)		
Immune effector cell-associated neurotoxicity syndrome (ICANS), *n* (%)	16 (59%)		27/27
Grade 1	10 (37%)		
Grade 2	3 (11%)		
Grade 3	2 (7%)		
Grade 4	1 (4%)		
Febrile neutropenia, *n* (%)	16 (59%)		27/27
Overall survival post-CAR T on 31 January 2026 (days)			27/27
Mean	324	275.85	
Median	277		
Overall survival post-CAR T among patients who died before 31 January 2026 (days)			9/9
Mean	299	274.69	
Median	213		
Hospital length of stay (days)			27/27
Mean	20	11.26	
Median	16		
Death by 31 January 2026, *n* (%)	9 (33%)		27/27

## Data Availability

The data presented in this study are available on request from the corresponding author due to restrictions imposed by the CIUSSS de la Capitale-Nationale in accordance with provincial privacy regulations. The minimal dataset cannot be shared publicly in order to protect patient privacy. Access may be granted to eligible researchers upon reasonable request and subject to completion of all required prerequisites, including, where applicable, a data use agreement. Data requests should be directed to the corresponding author.

## Abbreviations

The following abbreviations are used in this manuscript:

CAR	Chimeric Antigen Receptor T-Cell Therapy
CRS	Cytokine Release Syndrome
ICANS	Immune Effector Cell-Associated Neurotoxicity Syndrome
ECOG	Eastern Cooperative Oncology Group
CIUSSSCN	Centre Intégré Universitaire de Santé et de Services Sociaux de la Capitale-Nationale
GPI	Geriatric Prognostic Index
TAVI	Transcatheter Aortic Valve Implantation
GA-MDC	Geriatric Assessment-guided Multidisciplinary Clinics
ADL	Activities of Daily Living
IADL	Instrumental Activities of Daily Living
MoCA	Montreal Cognitive Assessment
OS	Overall Survival
GDS	Geriatric Depression Scale
DLBCL	Diffuse Large B-Cell Lymphoma
G-CSF	Granulocyte Colony-Stimulating Factor
SIOG	International Society of Geriatric Oncology
ESMO	European Society for Medical Oncology
